# Analysis of the Ki-67 Proliferation Index in Relation to Tumor, Node, and Metastasis (TNM) Stage in Patients With Oral Cavity Squamous Cell Carcinoma

**DOI:** 10.7759/cureus.63751

**Published:** 2024-07-03

**Authors:** Sathish Kumar, Jagjit Pandey, Shreekant Bharti, Satyanarayanan Senapati

**Affiliations:** 1 Surgical Oncology, All India Institute of Medical Sciences, Patna, Patna, IND; 2 Pathology, All India Institute of Medical Sciences, Patna, Patna, IND

**Keywords:** oral cavity prognostic marker, oral cavity malignancy, head and neck cancer surgery, ki 67 index, head and neck squamous cell carcinoma (hnscc), oral cavity cancer

## Abstract

Introduction

Squamous cell carcinoma (SCC) comprises more than 90% of malignant tumors of the oral cavity, accounting for up to 40% of all malignancies in South Asia. Despite the progress made in cancer management, the five-year survival rate for SCC has remained around 50%. To improve this survival rate, it is essential to understand the tumor’s biology at its core. In our study, the Ki-67 proliferation index of tumor cells was analyzed and correlated with the tumor stage, nodal stage, and tumor grade to determine the tumor’s biological aggressiveness.

Materials and methods

The study was conducted in a tertiary care center in South Asia from 2018 to 2022. A total of 50 adult patients with biopsy-proven oral cavity SCC were taken for analysis. The Ki-67 index was assessed in tumor cells using immunohistochemistry.

Results

Ki-67 was classified into two subcategories: <20% and >20%. Patients with an advanced T stage (T3-T4) have a greater chance of having a higher Ki-67 index (>20%), with p = 0.047. However, there is no statistically significant association between nodal status and tumor grade.

Conclusion

The Ki-67 proliferation index predicts the behavior of SCC lesions regarding tumor size and invasiveness.

## Introduction

Oral cavity carcinomas are the most common head and neck malignancies and originate from the squamous epithelium of the oral cavity. These carcinomas can be found in various locations, including the lip, mobile tongue, buccal mucosa, labial mucosa, floor of the mouth, gingiva, hard palate, and soft palate. The Asian population often suffers from oral cavity cancer due to habits such as betel quid or tobacco chewing. Squamous cell carcinoma (SCC) accounts for more than 90% of malignant tumors in the oral cavity [1] and up to 40% of all malignancies in South Asia. In the Indian and Sri Lankan populations, SCC of the buccal mucosa and gingiva buccal sulcus constitute 40% of oral SCC cases [2].

Despite advances in management, the patient survival rate has not significantly improved over the past 20 years, with five-year survival rates remaining around 50%. Genetic and epigenetic factors might play a significant role in disease recurrence [3]; however, there is currently no effective marker to predict SCC recurrence and prognosis. The molecular changes underlying tumor progression remain obscure and are the subject of numerous studies. Although tumor, node, and metastasis (TNM) classification is standard, it does not fully predict prognosis.

Identifying the molecular changes responsible for this process may contribute to a better understanding of tumor behavior. The Ki-67 antigen is a specific marker for proliferating cells [4]. Studies have demonstrated a highly significant correlation between Ki-67 staining and the degree of malignancy, with marked variations within different tumor grades, suggesting that Ki-67 staining may be useful in determining tumor behavior and prognosis [5].

This study evaluated the expression of the Ki-67 proliferation index to identify its role in oral cavity carcinogenesis, following possible correlations between different parameters, clinico-morphological analysis, and the selection of those with statistical significance. India, being a hub for oral cavity malignancies, provides highly authentic and reliable data. The Ki-67 index of tumor cells was analyzed alongside adjacent non-malignant cells and correlated with the tumor’s T stage, N stage, and grade.

## Materials and methods

An observational study was conducted at a tertiary care center in the eastern part of India from May 2018 to April 2022. A total of 50 adult patients with biopsy-proven oral cavity SCC were presented to the department of surgical oncology. Cases of oral cavity SCC that had been previously operated on and met the inclusion and exclusion criteria were analyzed. The inclusion criteria were patients over 18 years old with biopsy-proven, operable oral cavity SCC who had undergone surgery in the department of surgical oncology. The exclusion criteria included patients with non-squamous histology, recurrent tumors, metastatic tumors, or those who had received neoadjuvant chemotherapy.

The study utilized formalin-fixed paraffin-embedded tissue sections obtained from operated cases of oral cavity carcinomas and adjacent normal mucosa. The carcinomas were histologically graded based on the modified Broder’s grading system. Two independent pathologists reviewed the slides to validate the Ki-67 index. Invasive areas and adjacent nonmalignant mucosa were separately taken from gross specimens and confirmed via light microscopy.

Staining for immunohistochemistry was performed using the automated VENTANA Benchmark GX. Sections were taken from the representative blocks and transferred to Poly-L-Lysine coated slides, which were left to air dry for 12 hours. Each slide was then labeled with a barcode and subjected to immunohistochemistry staining with an antibody directed against the Ki-67 antigen. Nuclear expression of Ki-67 in tumor cells was interpreted as positive. The Ki-67 percent index was evaluated in 500 tumor cells from the area with the highest proliferation.

Antibody: Ki-67, Clone: MIB1, Monoclonal: Mouse, Company: Dako, Dilution: 1:50, Control: Normal tonsil mucosa

The Ki-67 results obtained in our study were within the 40% range; therefore, a new subset classification of Ki-67 was adopted: 0-20% were considered positive cells and > 20% were considered positive cells.

## Results

The mean age of the study participants was 50.8 ± 11.7 years. Predominant subsites included cancer of the lower gingivo buccal sulcus (42%), tongue (26%), buccal mucosa (20%), lip, and upper gingivo buccal sulcus, each accounting for about 6%. The majority of the study participants were males (88%), with only 12% being females.

All participants underwent oncological resection according to their clinical stage. TNM classification and Ki-67 index were calculated on post-operative histopathological specimens.

Tumor sizes were classified according to the standard National Comprehensive Cancer Network four-tier T stage system. It shows that most patients fell under the T4a subgroup (34.0%), followed by T2 (32.0%), T1 (18.0%), and T3 (16.0%). When tumor size was classified into two stages, early disease (T1-T2) and locally advanced disease (T3-T4), patients were equally distributed in both categories.

Among the study participants, most of the patients were nodal negative (52.0%), followed by N1 (18.0%), N2 (16.0%), and N3 (14.0%). Nodal status was almost equally distributed, with 52.0% in N0 and 48% in N+. Grade II (moderately differentiated) was the most common grade (78.0%) among study participants, followed by grade I (22.0%). None of the patients in our study had grade III tumors.

**Figure 1:**
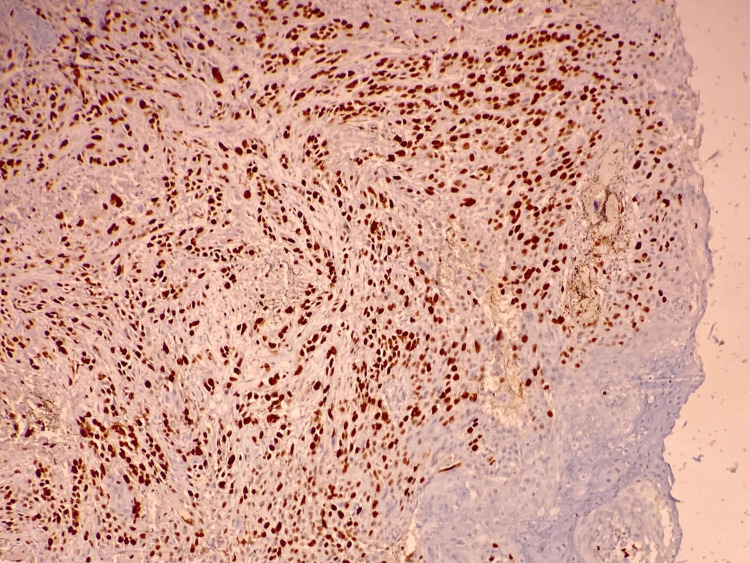
Ki-67 staining in tumor cells (40%).

The Ki-67 index was studied in all 50 participants. The value of Ki-67 in tumor cells is expressed in percentages from 2% to 40%, as depicted in Figure 1. The normality of the distribution of data was confirmed by visual inspection of the Q-Q plot and by the Shapiro-Wilk W test. Due to the unavailability of a standard classification of the Ki-67 index, a new subset classification was created based on our study’s results. Ki-67 was classified into only two subcategories: <20% and >20%. Most of the patients (76%) fell into the first category, and only 24% of them had a Ki-67 proliferation index value greater than 20%.

**Figure 2:**
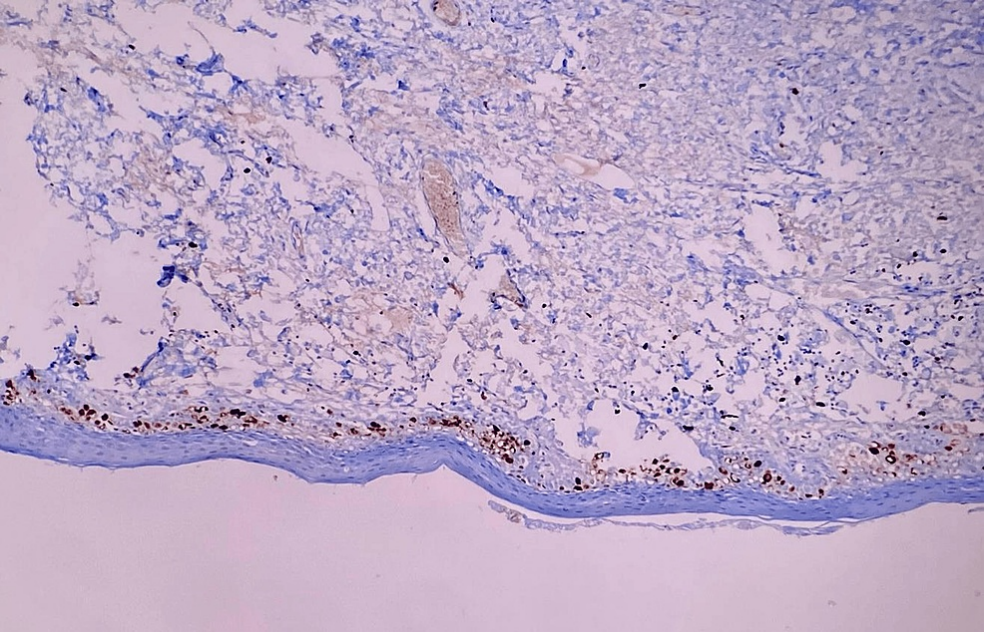
Ki-67 staining in normal tissue: brown cells (1%), confined only to the basal epithelium.

The Ki-67 value in adjacent normal tissue ranged from 1% to 3%, as depicted in Figure 2, highlighting the difference between Ki-67 in normal and tumor tissue. Despite the oral mucosa being an area of high proliferation, there is a significant difference in the proliferation index between normal cells and tumor cells.

On comparing T stage, N stage, and grade with the Ki-67 index, patients with an advanced T stage (T3-T4) had a greater chance (36%) of falling into the second Ki-67 category (>20%), with p = 0.047. This shows a statistically significant association between the T stage category and the Ki-67 tumor category. Patients with node positivity predominantly presented in the second Ki-67 category (>20%), and node-negative patients in the Ki-67 <20% category.

It has been observed that patients with Grade 1 have a greater chance (27.3%) of falling into the second Ki-67 category (>20%), compared with Grade 2 (23.1%). However, the value of the Chi-square calculated is 0.0828 (p-value), indicating that the difference is not statistically significant between the two groups.

## Discussion

Cell proliferation is a biological process of vital importance to all living organisms and is fundamental to both embryonic and post-embryonic existence. The control of this important biological process is thought to be lost in cancer, and many studies have reported that abnormal cell proliferation appears to be a precursor to, and may predict, tumorigenesis [6].

Ki-67 is a cell proliferation marker originally identified by Gerdes J et al. in 1980 [6]. It was named after its place of characterization in Kiel, Germany, and because the clone producing the antibody was grown in the 67th well of a tissue culture plate. The most prevalent analysis method of the Ki-67 antigen is immunohistochemical evaluation. Ki-67 nuclear antigen is expressed in certain phases of the cell cycle, namely S, G1, G2, and M phases, but is nonexistent in G0 [7,8]. Because of its high sensitivity and specificity in labeling cell proliferation in neoplastic tissues, it has been used to evaluate the aggressiveness of neoplasms [9].

This clinicopathological study aimed to correlate the Ki-67 proliferation index with the grade of the tumor, tumor size, and nodal burden in oral cavity cancer patients. Various factors contribute to the prognosis of oral cavity cancer patients, including histological variety, degree of differentiation, presence of metastatic adenopathy, perinodal extension, presence of perineural invasion, lymphovascular invasion, and the presence of residual malignant cells at the safety surgical margins, or margin status.

Considering all factors in an evaluation will confound and dilute the study’s design. Therefore, only the major prognostic factors, including grade, nodal status, and tumor size, were considered.

Molecular studies like the Ki-67 index for predicting the outcomes of head and neck malignancies represent a new area of interest. In our study, we aimed to correlate the Ki-67 index with T stage, N stage, and grade of the tumor to obtain meaningful data. Our study showed that a higher Ki-67 index was associated with a higher T stage. However, the correlation between a higher Ki-67 index and higher nodal stage and higher grade was not statistically significant.

Studies on the value of Ki-67 expression have shown conflicting results about the relationship between Ki-67 expression and survival. High Ki-67 expression showed shorter disease-free survival (DFS), although the results were not statistically significant, as reported by Kim SJ et al. [10].

A review article on the prognostic relevance of Ki-67 in different malignant tumors clearly demonstrated a statistically significant correlation between prognosis and Ki-67 [11]. This study shows that a Ki-67 proliferation index > 20% predicts a poor outcome in soft tissue sarcomas and serves as the sole independent predictor of overall survival.

Our study indicated that advanced T stages (T3-T4) have a greater likelihood of exhibiting higher Ki-67 levels. This corresponds to the study by Lopes VK et al., which revealed that T3 and T4 tumors showed a statistically significant relationship with Ki-67 immunoexpression. However, Ki-67’s relationship with age, gender, and tumor stage was not statistically significant [12].

We found no statistical difference between nodal status and tumor grade with the Ki-67 index. The negative correlation of the N stage and Ki-67 index in our study contradicts the findings by da Silva SD et al., who observed a significant positive association between patients with lymph node metastasis and Ki-67 expression [13].

In our study, well-differentiated (Grade 1) patients had a greater chance of having a higher Ki-67 index (> 20%). A study by Gonzalez-Moles MA et al. supported our findings by concluding that Ki-67 is significantly associated with well-differentiated tumors compared to moderately or poorly differentiated tumors [14]. Kearsley JH et al. investigated 42 fresh tumor samples of patients with SCC of the upper aerodigestive tract. In that study, the significance of the cellular expression of the Ki-67 antigen was analyzed among other tumor biological parameters in a prospective analysis of patients with SCCs of the head and neck. There was no statistically significant correlation with prognosis or other clinical parameters and proliferative activity [15].

Studies by Takkem A et al. and Gonzalez-Moles MA et al. also showed varying results when correlating Ki-67 with oral cavity carcinomas [16,17]. Numerous studies both support and contradict Ki-67’s role in oral cavity cancers. Our study shows that higher Ki-67 is associated with a higher T stage (T3-T4) and well-differentiated grade, but its association with the N stage is not significant. While this data may not be clinically relevant now, it paves the way for future introspection and correlation.

One limitation of our study is its small sample size. A large-scale, multi-institutional trial with a bigger sample size is needed to further accept or refute this proliferation marker.

Although the current literature provides varied opinions and data regarding the Ki-67 index in oral cavity cancers, our study underscores the importance of additional research with larger populations to explore its outcomes.

## Conclusions

To conclude, the Ki-67 proliferation index can be used to predict the behavior of SCC lesions with respect to tumor size and invasive tumors. Although our results conflict with current literature, they underscore the importance of the Ki-67 proliferation index in relation to tumor size. This study will encourage future colleagues to explore more molecular events that differentiate patients at risk for progression.
